# Effective pinning energy landscape perturbations for propagating magnetic domain walls

**DOI:** 10.1038/srep34517

**Published:** 2016-10-03

**Authors:** D. M. Burn, D. Atkinson

**Affiliations:** 1 Department of Physics, Imperial College London, London SW7 2BZ, United Kingdom; 2 Department of Physics, Durham University, Durham DH1 3LE, United Kingdom

## Abstract

The interaction between a magnetic domain wall and a pinning site is explored in a planar nanowire using micromagnetics to reveal perturbations of the pinning energetics for propagating domain walls. Numerical simulations in the high damping ’quasi-static’ and low damping ’dynamic’ regimes are compared and show clear differences in de-pinning fields, indicating that dynamical micromagnetic models, which incorporate precessionally limited magnetization processes, are needed to understand domain wall pinning. Differences in the micromagnetic domain wall structure strongly influence the pinning and show periodic behaviour with increasing applied field associated with Walker breakdown. In the propagating regime pinning is complicated.

Magnetization change in ferromagnets mediated by magnetic domain walls (DWs) is fundamental and widely utilized in many applications. Understanding of magnetic DW structure and magnetization processes has developed over many decades since the early works of Barkhausen^1^, Bloch^2^ and Landau and Lifshitz^3^. Significant new physical insight continues to emerge from experimental work^4^,^5^ and recent detailed theoretical treatment utilizing micromagnetic numerical simulations^6^,^7^,^8^. The advent of high-resolution lithographic methods allows individual DWs to be studied in magnetic nanostructures and led to the development of technology concepts for logic^9^ and memory^10^,^11^ applications, and the ability to detect^12^ and manipulate^13^,^14^,^15^,^16^ magnetic nanoparticles with DWs for use in drug delivery, nanofabrication and nanofluidics.

Significant interest in DW dynamics in recent years has focussed on the highly driven DW dynamics. Here a phenomenon known as Walker breakdown occurs where the motion of the DW adopts an oscillatory behaviour. Here DWs become pinned due to the evolution of non-equilibrium spin structures^17^,^18^ showing a stocastic nature when temperature effects are considered^8^. These studies contrast with earlier works where the de-pinning of a DW was treated as an energetics problem. Here we explore how the pinning effect on a DW varies from a statically pinned state to a propagating incident DW and reveal perturbations to the effective pinning energetic landscape as a function of the incident DW velocity.

Numerical simulations based upon micromagnetic formalisms are a key tool that provide physical insight into DW behaviour and are widely used to underpin the interpretation of experimental investigations of nanoscale magnetisation processes. Such numerical simulations solve the Landau-Lifshitz-Gilbert equation for an extended array of magnetic cells that gives the evolution of the magnetisation in response of ferromagnetic structure to internal demagnetising effects and applied magnetic fields. The damping parameter weights the time-dependent precessional decay in this relation and is important for accurately predicting time-dependent magnetization dynamics.

The physical origins of damping are complex and there are is some variation in the accepted values. In numerical modeling damping is treated as a phenomological constant representing a combination of intrinsic and extrinsic mechanisms. Also, it is common in micromagnetic studies, for computational efficiency, to arbitrarily increase the effective damping parameter to reduce simulation time without, it is assumed, significant loss of physical insight. Furthermore, numerical simulations of the interactions of DWs with pinning sites have not considered details of the micromagnetic state of dynamically propagating walls as they interact with a pinning site. Here the validity of the assumption of this ‘quasi-static’ regime is analysed and a series of new dynamic simulations show for the first time how the detailed micromagnetic structure of a DW arriving at a pinning site results in a complex range of pinning or non-pinning behaviour.

The use of constrictions^19^,^20^,^21^, notches^22^,^23^,^24^,^25^ and regions of reduced *M*_*S*_^26^ have previously been explored as mechanisms to controllably pin a DW at a known position within a nanowire structure. These ideas are based upon local modifications of the energy landscape of the wire to present a potential well or barrier to a DW. The DW interaction is determined by local energetic considerations, which depend upon the pinning site and, as is shown here, critically upon the the micromagnetic structure of the incident DW. DW interactions with different geometrical structures has been widely explored e.g. refs 27, 28, 29, 30, 31, 32, 33, 34.

In many previous micromagnetic studies the analysis of the quasi-static behaviour typically begins with the DW in close proximity to the pinning site and uses arbitrarily high damping^35^,^36^,^37^. Whilst these practical implementations improve computation efficiency, they may give simplistic results that do not represent the true complexity of the physical behavior. A fuller description needs to consider realistic damping dynamics and the micromagnetic structure of the DW as it arrives at the pinning site^38^,^39^,^40^,^41^. The structure of a propagating DW is sensitive to applied field, local roughness and stochastic thermal effects. Such considerations strongly influence the DW behaviour and already provide understanding of the propagation of DWs arriving at nanowire vertices^42^. Here the pinning behaviour is explored for an artificial pinning site and shown to be strongly influenced by the propagating nature and structure of the DW as it arrives at the pinning site.

## Physical Simulation Details

An extensive series of micromagnetic simulations were performed here using OOMMF^43^. Typical micromagnetic parameters for permalloy were used, i.e. *M*_*S*_ = 860 × 10^3^ A/m, *A* = 13 × 10^−12^ J/m and with zero magnetocrystalline anisotropy.

Numerical simulations in both the dynamic and quasi-static regime were investigated using 100 nm wide and 10 nm thick nanowire structures. These were divided into a mesh of cells 5 × 5 nm wide and 10 nm thick. The simulations were performed in a 0.5 *μ*m long simulation window where the magnetostatic effects of the nanowire ends were effectively removed by the inclusion of a plate of fixed magnetic charge^44^. A triangular notch feature was included in the center of the upper edge of the nanowire to act as a DW pinning site and this was parameterised by its depth.

The initial magnetisation configuration of the wire was two domains aligned along the wire axis separated by a head-to-head transverse DW positioned to the left of the pinning site. The DW structure was first allowed to relax at zero field to obtain an energy minimised micromagnetic configuration. An axial field was then applied in 1 Oe steps until the field energy enabled the DW to de-pin from the pinning site and propagate along the nanowire.

In the quasi-static regime, an artificially high damping parameter of *α* = 0.5 is commonly used to reduce simulation time^45^. Here, this approach was compared with simulations using a realistic value for the damping parameter, *α* = 0.01, using a torque stopping criteria of 1.0 degrees/ns. Significantly, these simulations set the scene for comparing the de-pinning behavior of static energetically minimised pinned DWs with that of dynamically propagating micromagnetic DWs when interacting with pinning features.

DWs in the dynamic regime were further investigated in 2.5 *μ*m long simulation windows where the initial separation between the DW and pinning site was varied and in each case an initial energy-minimised zero fieldstate in zero field was obtained. A spatially uniform magnetic field was then applied along the nanowire axis with a 1 ns rise-time ramp which reduced ringing in the micromagnetic structure. This protocol allowed a stable DW structure to be investigated within a reduced time and over a shorter propagation distance. Following the 1 ns ramp the field was maintained at a constant value until the DW either became pinned at the notch or continued to propagate to the end of the nanowire.

## Results and Discussion

**Figure 1:**
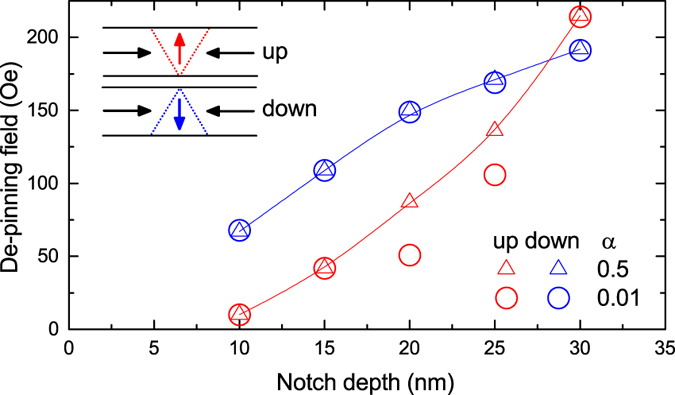
De-pinning field as a function of notch depth for a DW initially pinned at a notch in an energetically minimised state with damping parameters *α* = 0.01 and *α* = 0.5. The lines are a guide to the eye and the inserts show the geometry of the magnetisation in up- and down-chirality DWs respectively.

Initially, the de-pinning of an energetically minimised DW structure from a notch was investigated in the quasi-static regime with an increasing applied field supplying energy to overcome the pinning. Figure 1 shows the de-pinning fields for both up- and down-chirality DWs interacting with a range of notch depths simulated with *α* = 0.01 and 0.5.

As expected, with increasing notch depth the DWs experience stronger pinning, requiring a higher field to de-pin from the notch. There is also a clear difference in the pinning of DWs with different chiralities from shallower notches. In this regime, the size of the notch is comparable with the finer details of the micromagnetic structure where one end of the DW having a 180° spin rotation over a much shorter distance than the other giving it a distinctive triangular shape. The chirality of the DW then determines which end of the DW experiences the pinning effect of the notch. Analysis shows the static modelling of DW de-pinning behavior is not signficantly affected by value of the damping parameter, i.e. it is reasonable to use an artificially high damping parameter (*α* = 0.5) to investigate energy minimised micromagnetic behaviour in the quasi-static regime.

Critically, this quasi-static analysis is physically simplified and it is now shown here that a new and significantly more complex physical description of DW pinning is obtained with dynamical simulations that take into account the dynamical micromagnetic structure of an incident DW.

**Figure 2:**
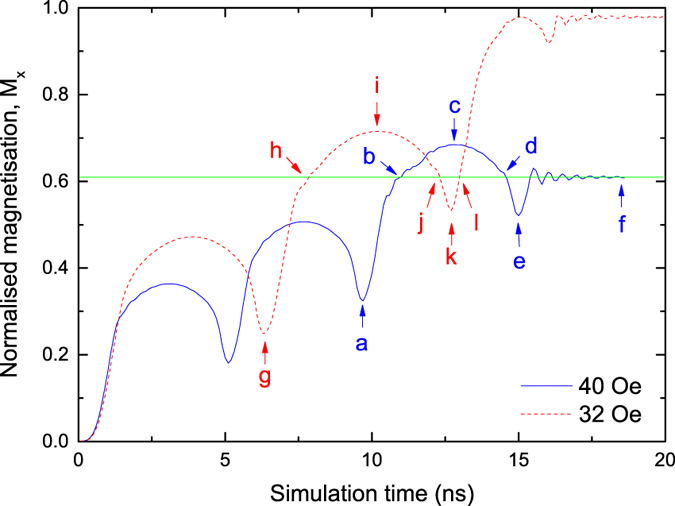
Magnetisation as a function of time for a DW incident upon a 15 nm notch on the top edge of the nanowire with an initial separation of 750 nm. The change in magnetisation occurs due to DW propagation in either a 32 Oe or 40 Oe driving field.

The interactions of dynamically propagating DWs with a pinning site are analysed by following the time-evolution of the nanowire magnetisation. With a magnetic field the DWs propagate along the nanowire, increasing the size of the domain with magnetisation aligned with the field and increasing the net magnetisation. Figure 2 shows an example of the DW behaviour for an initial DW with up-chirality propagating at two different fields of 32 Oe and 40 Oe.

At both 32 and 40 Oe DW propagation was in the Walker breakdown regime^46^. Here the motion of the DW is periodically interrupted by the formation of an anti-vortex core which traverses the nanowire width, reverses the DW chirality and leads to a reduction in average DW velocity. In both cases DW motion continues up to the position of the notch, represented by the horizontal line on the figure at *M*_*x*_ ≈ 0.6.Then the DW behaviour depends upon the applied field and is counterintuitive. At 40 Oe field the DW becomes pinned at the notch, but at 32 Oe the DW propagates past the notch to the end of the nanowire.

A common understanding of the interaction between the DW and notch is based on the pinning potential of the notch. With increasing applied field the Zeeman energy eventually overcomes the pinning potential and the DW is de-pin. However, Fig. 2 shows DW propagation at 32 Oe, but at a higher field and greater Zeeman energy, the DW is pinned. Note also that for both fields in Fig. 2, a significant initial increase in magnetisation occurs beyond the position of the notch, indicating that in both cases the DW initially propagates beyond the notch, prior to retrograde motion which returns the DW to the notch which leads to the interactions that determine subsequent pinning or propagation.

**Figure 3:**
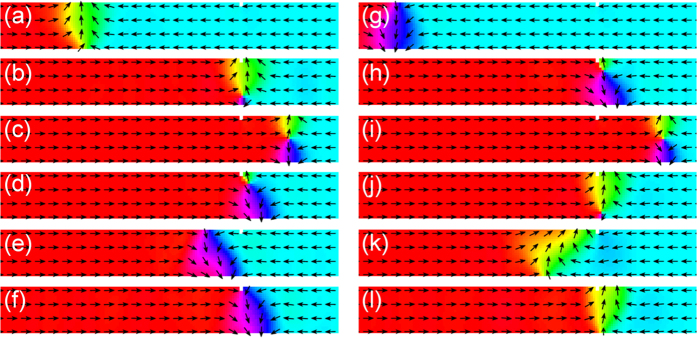
Snapshots of the micromagnetic spin configuration of a DW during its interaction with a notch. (**a**–**f**) show the behaviour at 40 Oe whilst (**g**–**l**) show behaviour at 32 Oe.

Snapshot images of the micromagnetic structure during DW propagation are shown in Fig. 3 and correspond to the labeled points in Fig. 2. For the two different fields the DW is at different stages of the Walker breakdown cycle when it first interacts with the notch (Fig. 3b,h) and most significantly the two DWs have opposite chirality. In both cases the micromagnetic structures are such that the DW initially continues past the notch and an anti-vortex core nucleates in the wall structure and traverses the wire width (Fig. 3c,i). Retrograde DW motion occurs as the anti-vortex core is annihilated (Fig. 3d,j) and the chirality of the subsequent transverse DW is reversed. In this case the down-chirality DW (Fig. 3f) becomes pinned at the notch but the up-chirality wall (Fig. 3l) continues to propagate along the wire at the lower field.

**Figure 4:**
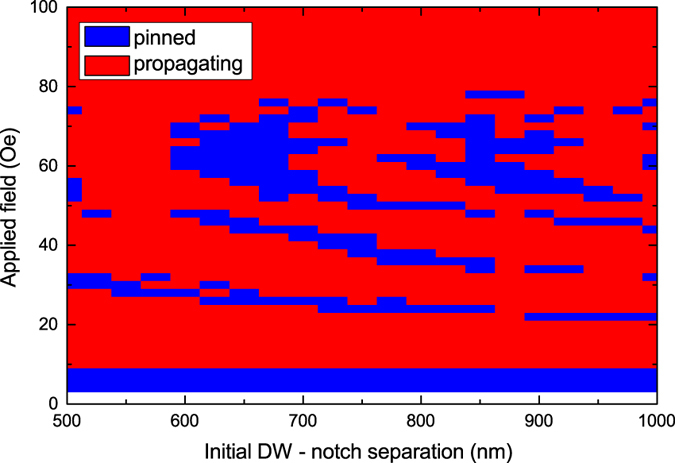
Phase diagram showing the final state of the DW, pinned or propagating as a function of the applied field and initial DW - notch separation.

The field dependence of the pinning behaviour was determined from the final state of the system (pinned or propagating DW) as a function of both the applied field and the initial DW-to-notch separation distance. This allows the DW pinning interaction to be investigated for a range of dynamically different DW micromagnetic states as the exact structure of wall on arrival at the notch will be different dependent upon the path-length traveled, this also effectively relates to different DW velocities. Figure 4 shows the final state of an initial up-chirality DW interacting with a 15 nm deep notch on the upper edge of a planar nanowire as a function of the initial DW-notch separation and magnetic field. Figures for additional notch geometries are shown in the supplementary material. Even though the details vary, a consistent non-monotonous pinning probability with field is observed. It is expected that sample to sample variations would show a similar behaviour.

For a 100 nm wide wire with a 15 nm deep notch, below 10 Oe the DW is always pinned, and up to 20 Oe the DW consistently propagates through the notch without becoming pinned. In both cases the behaviour does not depend on the initial DW to notch separation and this regime represents the propagation of a DW below the Walker field. The difference between the pinned and propagating DWs relates to the energy barrier arising between the DW micromagnetic structure and the geometrical structure of the notch. Note that this barrier appears lower than for the same notch in Fig. 1 which will be discussed in more detail later.

In the high field regime above 80 Oe, the figure also shows consistent propagation of the DW where the field is sufficient to overcome the pinning potential at the notch for any micromagnetic structure the DW can adopt.

However, for the important intermediate fields the pinning behaviour is complex, where DWs are either pinned or able to propagate through the notch depending upon a combination of the applied field and the initial separation between the DW and the notch. Both of these factors determine the precise micromagnetic DW structure as it arrives at the notch, which in turn leads to variations in the pinning interaction between the DW and the notch. The striped pattern in the figure shows a periodic dependence of the pinning behaviour as the applied field is increased. This becomes more pronounced with greater initial DW-notch separations and curvature of the striped pattern is observed with greater fields. For the given notch the pinning behavior corresponds to down-chirality DWs interacting with the notch and the stripes represent the greater number of Walker breakdown transitions in DW chirality as the field is increased.

**Figure 5:**
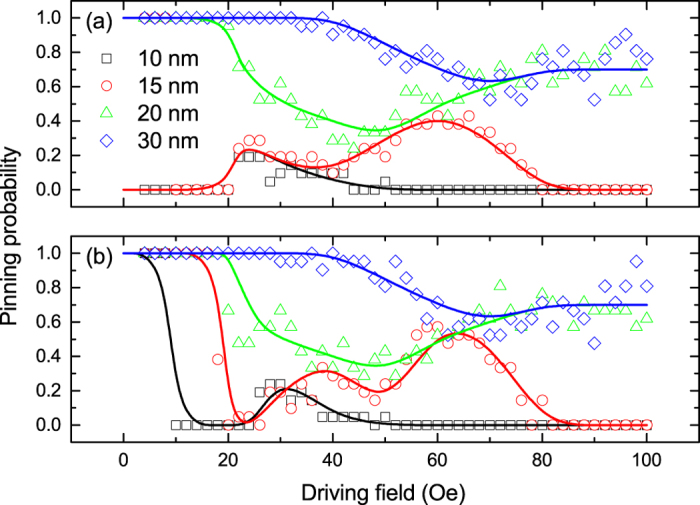
Effective pinning probability as a function of applied field for DWs with (**a**) up- and (**b**) down-chirality in a nanowire with various different notch structures. The lines are a guide to the eye.

The results thus far have described up-chirality DWs approaching a 15 nm notch on the upper edge of the nanowire. However, the notch geometry has an important effect on the pinning of the DW as well as the dynamic state of the DW micromagnetic structure. The results in Fig. 4 can be more easily compared for a variety of notch structures by averaging over the range of different micromagnetic structures found as a function of separation. This gives an effective probability that a DW becomes pinned at the notch as a function of field. Figure 5 shows this field dependence of the pinning for different notches and with initial chirality up and down.

For both DW chiralities pinning is consistent at low fields, where deeper notches show 100% pinning regardless of the micromagnetic DW state. At the highest fields here, the DWs are not pinned by the smaller notches but have a high probability of being pinned at deeper notches. For intermediate fields all the notches display a pinning probability that takes on a complex profile. For intermediate to high fields these profiles are very similar for both DW chiralities and differences only become significant at the lower fields. Below the Walker field, the up-chirality DWs are able to pass the smallest notches at low fields, whilst, the down-chirality DWs experience much stronger pinning, which agrees with early experimental work^11^.

The fields required to de-pin a stationary DW (Fig. 1) are considerably larger than the fields at which the pinning probability drops from 100% for a propagating DW (Fig. 5). This difference between the pinning effect of DWs incident upon the same notch gives physical insight into the differences in behaviour between the dynamic and quasi-static regimes.

This difference can be understood in terms of the difference in DW micromagnetic structure between the two cases. In the quasi-static case the DW structure maintains an energetically favourable configuration around the notch for each applied field, which increases the de-pinned field required to escape from the notch. In the dynamic case, the system has insufficient time for the DW structure to adopt a lowest energy configuration in the vicinity of the notch. Instead the DW arrives at the notch in a configuration given by the propagation energetics and precessional dynamics of spins in the wall and this structure does not experience the same pinning effect of the notch as a quasi-statically arriving wall.

## Conclusions

Numerical micromagnetic simulations were used to investigate DW pinning in both dynamic and quasi-static modeling regimes. These zero-temperature models of structures with uniform magnetic properties give insight into the physical behaviour of DWs that may become significant in experimental work and through the development of novel technological devices. DW micromagnetic structure, which varies significantly throughout the Walker breakdown cycle, is found to strongly influence the pinning behaviour which is not, therefore, simply based on the Zeeman energy being in excess of a single de-pinning field. Furthermore, DWs can propagate through pinning features at fields considerably lower than the de-pinning field derived from a static DW interaction, where the DW has relaxed into an energetically minimised state. This new physical insight into the pinning behaviour of dynamically propagating DWs will be useful in the development of new technological devices based on the manipulation of magnetic DWs.

## Additional Information

**How to cite this article**: Burn, D. M. and Atkinson, D. Effective pinning energy landscape perturbations for propagating magnetic domain walls. *Sci. Rep.* **6**, 34517; doi: 10.1038/srep34517 (2016).

## Supplementary Material

Supplementary Information
