# Corrigendum: Effective pinning energy landscape perturbations for propagating magnetic domain walls

**DOI:** 10.1038/srep37048

**Published:** 2016-11-10

**Authors:** D. M. Burn, D. Atkinson

Scientific Reports 6: Article number: 3451710.1038/srep34517; published online: 10 03 2016; updated: 11 10 2016

The Acknowledgements section in this Article was omitted. The Acknowledgements section should read:

“The authors gratefully acknowledge funding from the UK EPSRC for the PhD studentship [EP/P504686/1 and EP/P505186/1] of DMB”.

