# Supplementary material for: Effective pinning energy landscape perturbations for propagating magnetic domain walls
Source: Sci Rep. 2016 Oct 3;6:34517. doi: 10.1038/srep34517 (PMC5046081; doi:10.1038/srep34517)

## Supplementary material

# Effective pinning energy landscape perturbations for propagating magnetic domain walls

D.M. Burn[1] and D. Atkinson[2]

1. Department of Physics, Imperial College London, London SW7 2BZ, United Kingdom
2. Department of Physics, Durham University, Durham DH1 3LE, United Kingdom

# Dynamic pinning with various notch geometries

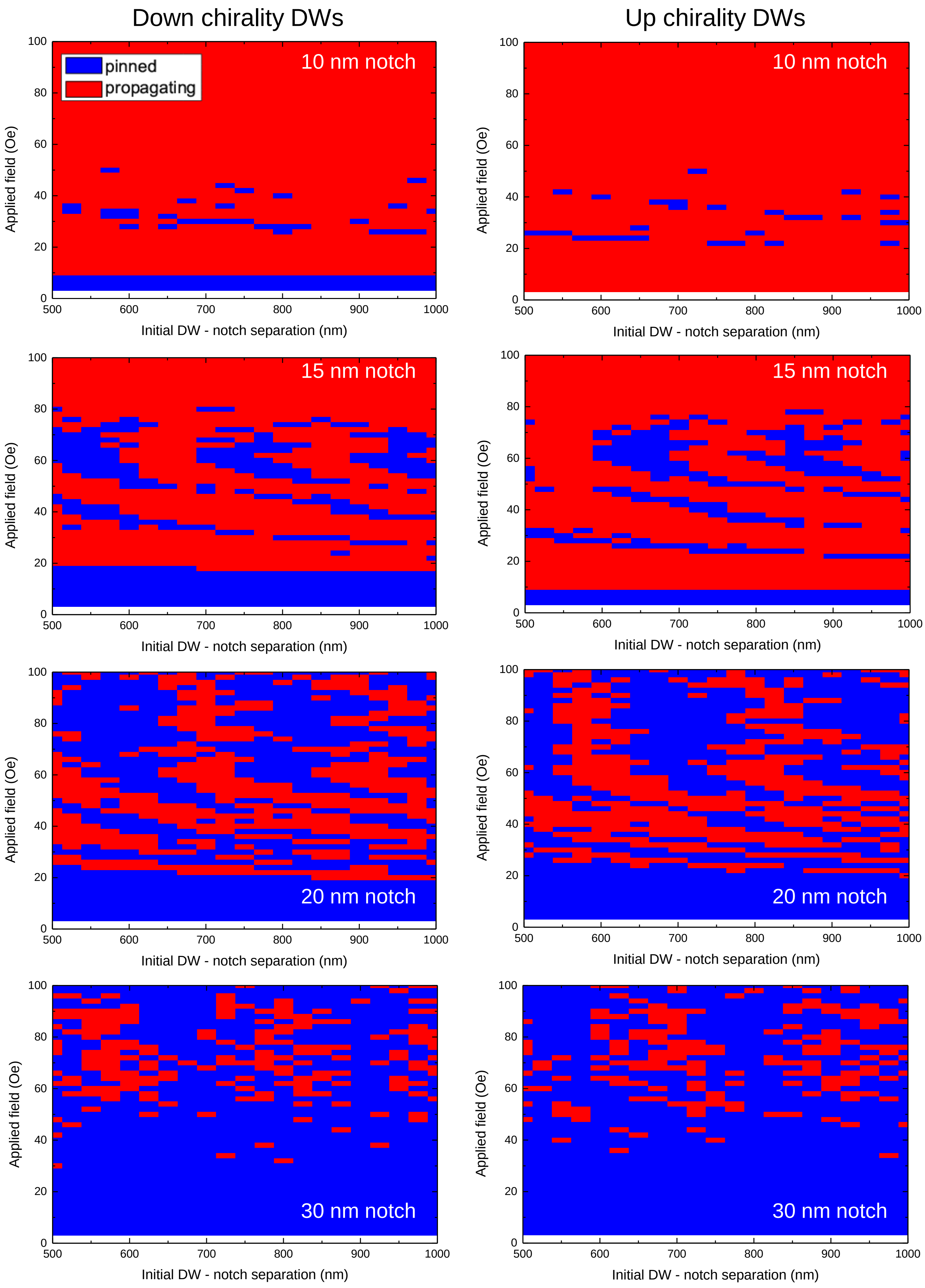

Supplement: Supplementary Information [file srep34517-s1.pdf]
